# Infliximab abrogates adenine-induced chronic kidney disease via modulation of the MAPK/JNK/ASK signaling pathway in rats

**DOI:** 10.1007/s00210-023-02585-4

**Published:** 2023-07-04

**Authors:** Mahitab M. Nageeb, Aliaa Talaat, Samar M. Reda, Ghada A. Elsammak

**Affiliations:** 1 Department of Pharmacology, Faculty of Medicine, Zagazig University, Zagazig, Egypt; 2 Department of Medical Biochemistry, Faculty of Medicine, Zagazig University, Zagazig, Egypt; 3 Department of Histology and Cell Biology, Faculty of Medicine, Zagazig University, Zagazig, Egypt

**Keywords:** Chronic kidney disease, Adenine, Infliximab, Oxidative stress, Apoptosis, Inflammation

## Abstract

Chronic kidney disease (CKD) is a prominent cause of death worldwide. Infliximab is one of the anti-TNF-α; herein, we studied the effect of infliximab on adenine-induced CKD. To inspect the role of infliximab, either ameliorative or curative, on CDK induced with adenine. Thirty Wistar albino rats were separated into five groups of 6 rats’ each: rats of group Ι were kept as control given saline, rats of group II were treated with infliximab (5 mg/kg, i.p.) for 5 weeks, rats of group ΙΙΙ (the diseased group) had an adenine containing diet (0.25% W/W in feed) for 5 weeks, rats of group ΙV (the ameliorative group) had an adenine-containing diet and infliximab (5 mg/kg, i.p.) for 5 weeks simultaneously, and rats of group V (the curative group) had adenine containing diet then a single dose of infliximab (5 mg/kg, i.p.) was given in the 6th week. Infliximab treatment revealed a decrease in the plasma levels of urea, creatinine, NGAL, and MDA with a substantial increase in TAC. Also, inflammatory mediators such as IL-6 and NF-κB were significantly decreased with the down-regulation of the ASK1/MAPK/JNK pathway. Caspase 3 was downregulated. Also, infliximab treatment exhibited improvement in the histological and immunohistochemical kidney changes. Through its involvement in reducing oxidative stress, inflammation, and apoptosis, infliximab has an ameliorative and curative effect on CKD induced with adenine.

**Supplementary information:**

The online version contains supplementary material available at 10.1007/s00210-023-02585-4.

## Introduction

Chronic kidney disease (CKD) is one of the momentous long-term problems seen all over the world (Hsu and Powe 2017). It is a substantial contributor to the global illness burden, affecting more than 200 million people globally (Gori et al. 2021), and there is currently no drug available that can be used to improve kidney performance in CKD patients. Current clinical approaches are primarily limited to slowing down disease development in end-stage renal failure conditions, where dialysis or kidney replacement is the only management choice (Cai et al. 2018). Herein, it is necessary to find new therapies to abate the effects of the disease or even postpone the regression in the function of the kidney.

Kidney disease is caused by a variety of factors, including systemic angiopathy caused by diabetes, hypertension, congenital diseases, and glomerulonephritis. When the disease advances to CKD, fibrosis of the renal interstitium is usually detected, leading to terminal renal failure with a poor prognosis (Ito et al. 2022).

Understanding the molecular mechanisms of CKD is compulsory for dwindling organ loss. As reported, inflammatory cytokines are likely to have a role in the evolution of kidney disease by inducing chronic inflammation as well as oxidative stress and programmed cell death (Zoccali et al. 2017). So, reducing inflammation and oxidative stress is an essential strategy to reduce CKD progression.

Yokozawa et al. (1986) described the adenine-induced model of CKD, which is regarded as one of the most widely used fortunate models (Yokozawa et al. 1986). Adenine and its metabolite, 2,8-dihydroxyadenine, precipitate and crystallize within the renal tubules, leading to blockage, ischemia, fibrosis, and growth retardation. Furthermore, compared to the 5/6 nephrectomy model of CKD, this model is simple to employ, has a low death rate, requires no operating expertise, and has an advanced character that mimics individual CKD (Diwan et al. 2013).

Infliximab is a chimeric human murine monoclonal antibody (165 kDa) that binds to soluble and transmembrane-bound tumor necrosis factor (TNF-α), creating stable immune complexes. It is used in clinical practice because of its enormous size and the fact that it does not penetrate the blood–brain barrier when administered systemically, so it targets primarily peripheral TNF- α (Poutoglidou et al. 2022). In patients being affected by sarcoidosis or arthritis of a rheumatoid nature, drugs working against TNF-α showed promising results (Raftery et al. 2012). They have also been proposed as a therapy for Alzheimer's disease cognitive impairment (Cheng et al. 2014).

## Materials and methods

### Materials

#### Experimental animals

Male Wistar albino rats weighing 150–200 g were bought from the animal unit ZSMRC at Zagazig University’s Faculty of Medicine. Rodents were given free access to a regular rat pellet chow diet and water.

#### Drugs

Adenine was obtained from Sigma Aldrich Co. LLC, St. Louis, MO, the USA with ≥ 97% purity. Infliximab powder ≥ 97% purity (Sigma Aldrich Co. LLC, St Louis, MO, USA) was further assessed in our laboratory by HPLC.

Saline was used as a vehicle for infliximab powder (10 mg/ml) for intraperitoneal (i.p.) administration.

### Methods

#### Experimental design

Thirty Wistar albino rats (9–10 weeks old, originally weighing about 150–200 g) were sheltered in a room with a temperature of 22 ± 2 °C, a relative humidity of about 60%, a 12-h light–dark cycle (lights on at 6:00 and off at 18.00), and fed ad libitum a standard pellet chow diet containing 0.85% phosphorus, 1.12% calcium, 0.35% magnesium, 25.3% crude protein, and tap water and were then placed into 5 groups (6 rats’ each) at random:

- Group Ι (control): normal rats received saline as a vehicle (i.p.) daily for 5 weeks with a normal diet.
- Group ΙΙ (infliximab): rats were treated with infliximab (5 mg/kg, i.p.) (Mohamad et al. 2022) for 5 weeks.
- Group ΙΙΙ (diseased): rats were fed an adenine-containing diet (0.25% W/W in feed) for 5 weeks (Ali et al. 2018).
- Group ΙV (ameliorative): rats were fed an adenine-containing diet (0.25% W/W in feed) for 5 weeks and infliximab was given at a dose of (5 mg/kg, i.p.) for 5 weeks along with the adenine diet (Mohamad et al. 2022)
- Group V (curative): rats were fed an adenine-containing diet (0.25% W/W in feed) for 5 weeks, then a single dose of infliximab (5 mg/kg, i.p.) was given at the 6th week (Mohamad et al. 2022).

Rats’ follow-up was performed throughout the experiment by observing urine output and their weight, and at the end of the experiment, rats were anesthetized using thiopental sodium (5 mg/kg, i.p.), and blood was withdrawn from the retro-orbital spaces of all rats and centrifuged for serum separation. The left kidney from all rats was removed, washed with ice-cold saline, and stored at − 80 °C for gene expression analysis; the right kidney from all rats was fixed for histopathological examination.

### Biochemical study

#### Detection of total antioxidant capacity (TAC)

The detection of total antioxidant capacity (TAC) was measured spectrophotometrically in the kidney homogenate. The determination of the antioxidative capacity is performed by the reaction of antioxidants in the sample with a defined amount of exogenously provided hydrogen peroxide (H2O2).The antioxidants in the sample eliminate a certain amount of the provided hydrogen peroxide. The residual H2O2 is determined colorimetrically by an enzymatic reaction, which involves the conversion of 3,5,dichloro –2– hydroxybenzenesulphonate to a colored product (Biodiagnostic kit,CAT.NO: TA 25 13) (Koracevic et al. 2001).

#### Evaluation of malondialdehyde (MDA)

A marker of lipid peroxidation was measured spectrophotometrically in the kidney homogenate. Thiobarbituric acid (TBA) reacts with malondialdehyde (MDA) in the acidic medium at a temperature of 95 °C for 30 min to form a thiobarbituric acid reactive product. The absorbance of the resultant pink product can be measured at 534 nm (Biodiagnostic Kit,CAT.NO: MD 25 29) (Satoh 1978).

#### Measurement of serum urea

The method is based on the following reaction:

$$\begin{array}{c}\mathrm{Urease}\\ \mathrm{Urea}+\mathrm{H}2\mathrm{O}\to 2\mathrm{ NH}3+\mathrm{CO}2\end{array}$$

The ammonium ions formed were measured by the Berthelot reaction. The blue dye indophenol product reaction absorbs light proportional to the initial urea concentration (Biodiagnostic Kit, CAT.NO: UR 21 10) (Fawcett and Scott 1960).

#### Measurement of serum creatinine

Creatinine was measured according to the principle of that it forms a colored complex with picrate in an alkaline medium (Biodiagnostic Kit, CAT.NO: CR 12 50) (Schirmeister et al. 1964).

#### Evaluation of serum IL-6, NF-KB, and NGAL

Il-6, NF-KB, and neutrophil gelatinase-associated lipocalin (NGAL) were measured in serum using ELISA kits (MyBioSource) (CAT NO.MBS355410, CAT NO. MBS287521, CAT NO.MBS260195), respectively, according to the manufacturer’s instructions.

#### Gene expression analysis

Total RNA was extracted according to the manufacturer’s recommendations using a Qiagen kit. The extracted RNA was reverse transcribed using the QuantiTect Reverse Transcription Kit as directed by the manufacturer. A 20 µL reaction mixture including 5 µLcDNA template, 10 µLEva Green mix (Jena Bioscience), and 100 pmol/l primers was used to amplify specific RNA. The primers are shown in Table 1.

**Table 1:** Primers’ sequences of MAPK, JNK, ASK1, Caspase 3, and GAPDH

Target gene	5′ → 3′ primer sequence	Tm (_C)
MAPK	F: AGTGGCTGACCCTTATGAC; R: CACAGTGAAGTGGGATGGA	55
JNK	F: ATTTGGAGGAGCGAACTAAG; R: ATTGACAGACGGCGAAGA	60
ASK1	F: GCCGTGCTGGACCGTTTTTAC R: GTGAGGCGTGATGTAAATAGGAAGC	60
Caspase 3	F: GTGGAACTGACGATGATATGGC R: CGCAAAGTGACTGGATGAACC	60
GAPDH	F: GTCGGTGTGAACGGATTTG R: CTTGCCGTGGGTAGAGTCAT	57

Amplification was carried out using a real-time polymerase chain reaction (PCR; Strata Gene Mx3005P-qPCR System). Glyceraldehyde-3-phosphate dehydrogenase (G3PDH) is expended as a housekeeping gene. The PCR cycling parameters were as follows: initial heating at 95 °C for 2 min, followed by denaturation at 95 °C for 15 s, annealing at the corresponding temperature in Table 1 for 15 s, and extension at 72 °C for 30 s (40 cycles), and final extension at 72 °C for 10 min. The 2-CT approach (Livak’s method) was used to calculate relative changes in gene expression (Livak and Schmittgen 2001).

## Histopathological methods

### Light microscope technique

After 24 h after the final dose, Thiopental Sodium was used for rats’ anesthesia, then rats were sacrificed and carefully dissected, and specimens of the kidneys were processed for light microscopic examination.

Formal saline was used to fix kidney specimens from all animal groups. The specimens were prepared for paraffin sections with a thickness of 7 m. Hematoxylin and eosin (H&E) were used to show the histological structure of the sections, and Mallory’s trichrome stain was used to reveal collagen fibers (Bancroft and Layton 2012).

### Immunohistochemical technique

For immunohistochemical staining, sections of 5-µm thickness were dewaxed, rehydrated, and rinsed with phosphate-buffered saline (PBS) and then incubated with PBS containing 10% normal goat serum. Sections were incubated with rabbit polyclonal antibody against TNF-α (ab6671, Abcam, Cambridge, Massachusetts, USA) and mouse anti-desmin (Lab Vision Corp, Inc/Lab Vision, Fremont, USA) (Pollock et al. 1995) night long in a humid chamber at 4 °C, followed by 60 min at room temperature incubation with biotinylated goat anti-rabbit IgG.

The sections were incubated with a streptavidin–biotin–horseradish peroxidase complex for another 60 min. As a chromogen, 3,3′-diaminobenzidine (DAB) hydrogen peroxide was used to visualize immunoreactivity, and slices were counterstained with Mayer’s hematoxylin. By omitting the primary antibodies, negative control sections were ready (Ramos-Vara et al. 2008).

### Morphometric study

At a magnification of × 400, the Image Analyzing Unit of the Pathology Department, Faculty of Dentistry, Cairo University, Egypt, used the Leica Qwin 500 image analyzer computer system (Leica Ltd, Cambridge, UK) to measure the area percentage of collagen fibers and the area percentage of immunoreaction for desmin and TNF-α. Using the interactive measure menu, the area percentage was calculated.

The measurement frame was chosen to have a standard area of 118476.6 mm^2^ so that the brown positive immunological reaction could be seen and the blue binary color could be measured. This was performed in 5 non-overlapping fields of 5 different sections of 5 rats in every group.

### Statistical analysis

The data were analyzed on a computer using the Statistical Package of Social Services version 24 (SPSS). A One-way analysis of variance (ANOVA) was used. The data are displayed in tables, and continuous quantitative variables were expressed as mean and SD.

After verifying for normality, appropriate statistical tests of significance were performed. When the significant probability was less than 0.05 (*P* 0.05), the results were declared statistically significant. *P*-values less than 0.001 were deemed highly statistically significant (HS), whereas *P*-values less than 0.05 were deemed statistically insignificant (NS) (Dawson-Saunders and Trapp 2001).

## Results

### Biochemical results

As shown in Table 2, treatment with adenine revealed a significant (*P* < 0.05) increase in the tissue level of MDA and significant (*P* < 0.05) decrease in the level of TAC compared to the control group. In the contrary, infliximab treatment showed a significant (*P* < 0.05) decrease in the level of MDA and a significant (*P* < 0.05) increase in TAC tissue level in the curative and the ameliorative groups in comparison with the diseased group.

**Table 2:** Effect of infliximab on TAC and MDA levels on adenine induced CKD in adult male albino rats

Mean ± SD	Group I (control)	Group II (infliximab)	Group III (diseased)	Group IV (ameliorative)	Group V (curative)
TAC (nmol/mg protein)	57.41 ± 2.93	56.15 ± 2.69	25.71 ± 3.17 ^*#^	51.78 ± 0.36 ^*&#^	42.95 ± 2.69 ^*&#$^
MDA (nmol/mg protein)	25.16 ± 2.12	24.76 ± 2.15	111.26 ± 9.20 ^*#^	33.01 ± 2.86 ^*&#^	47.99 ± 2.18 ^*&#$^

*TAC* total antioxidant capacity, *MDA* malondialdehyde

Data represent mean ± SD, significant *P*-value < 0.05

^*^Significant to the control group

^&^Significant to the diseased group

^#^Significant to the infliximab group

^$^Significant to the ameliorative group

Treatment with adenine indicated a significant (*P* < 0.05) increase in the levels of serum urea, creatinine, and NGAL compared with the control group. However, infliximab treatment showed a significant (*P* < 0.05) decrease in serum urea, creatinine, and NGAL levels in the curative and the ameliorative groups compared with the diseased group (Table 3).

**Table 3:** Effect of infliximab on urea, creatinine, and NGAL serum levels on adenine induced CKD in adult male albino rats

Mean ± SD	Group I (control)	Group II (infliximab)	Group III (diseased)	Group IV (ameliorative)	Group V (curative)
Urea (mg/dl)	47.23 ± 6.47	51.68 ± 2.86	112.68 ± 7.59^*#^	65.40 ± 1.66^*&#^	63.75 ± 3.42^*&#^
Creatinine (mg/dl)	0.14 ± 0.02	0.14 ± 0.01	1.9 ± 0.18^*#^	0.341 ± 0.02^*&#^	0.63 ± 0.09^*&#^
NGAL (pg/ml)	215.25 ± 2.44	216.02 ± 2.12	402.38 ± 88.11^*#^	240.20 ± 6.37^*&#^	262.12 ± 15.48^*&#^

*NGAL* neutrophil gelatinase-associated lipocalin

Data represents mean, significant *P*-value < 0.05

^*^Significant to the control group

^&^Significant to the diseased group

^#^Significant to the infliximab group

Treatment with adenine indicated a significant (*P* < 0.05) increase in the tissue levels of IL-6 and NF-κB compared with the control group. Infliximab treatment showed a significant (*P* < 0.05) decrease in IL-6 and NF-κB levels in the curative, and the ameliorative groups compared with the diseased group as displayed in Table 4.

**Table 4:** Effect of infliximab on IL6 and NF-κB levels on adenine induced CKD in adult male albino rats

Mean ± SD	Group I (control)	Group II (infliximab)	Group III (diseased)	Group IV (ameliorative)	Group V (curative)
IL6 (pg/ml protein)	36.48 ± 1.74	35.12 ± 2.72	130.83 ± 6.81^*#^	49.55 ± 1.24^*&#^	51.75 ± 3.38^*&#^
NF-κB (pg/ml protein)	88.15 ± 1.22	88.13 ± 1.18	274.38 ± 16.17^*#^	99.24 ± 5.20^*&#^	153.67 ± 15.48^*&#^

*IL-6* interleukin 6, *NF-κB* nuclear factor kappa B

Data represents mean, significant *P*-value < 0.05

^*^Significant to the control group

^&^Significant to the diseased group

^#^Significant to the infliximab group

Treatment with adenine showed a significant (*P* < 0.05) increase in caspase 3 mRNA expression compared with the control group. In the contrary, infliximab treatment showed a significant (*P* < 0.05) decrease in caspase 3 mRNA expression in the curative and the ameliorative groups compared with the diseased group (Table 5).

**Table 5:** Effect of infliximab on caspase 3 gene expression on adenine induced CKD in adult male albino rats

Mean ± SD	Group I (control)	Group II (infliximab)	Group III (diseased)	Group IV (ameliorative)	Group V (curative)
Caspase 3	1.16 ± 0.36	1.17 ± 0.38	9.76 ± 0.82 ^*#^	2.94 ± 0.14 ^*&#^	3.96 ± 0.09^*&#^

Data represents mean, significant *P*-value < 0.05

^*^Significant to the control group

^&^Significant to the diseased group

^#^Significant to the infliximab group

**Fig. 1:**
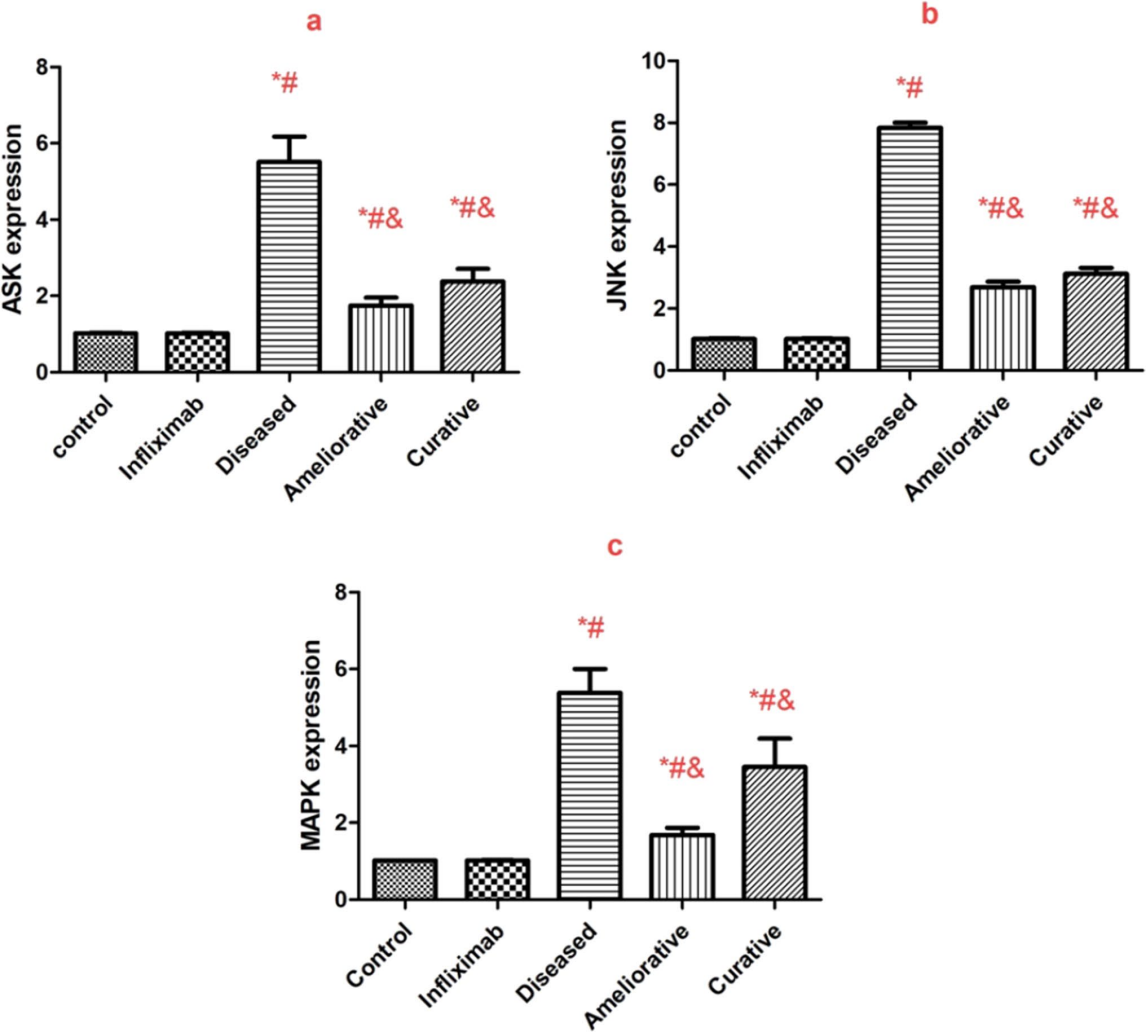
Gene expression of ASK (Apoptosis signal-regulating kinase) (**a**), Jun N-terminal kinase JNK (**b**), and mitogen-activated protein kinase (MAPK) (**c**) increased significantly with adenine treatment. Infliximab administration showed a significant decrease in MAPK, ASK & JNK expression as an ameliorative or curative agent. Data represents mean ± SD, *significant to the control group, &significant to the diseased group, #significant to the infliximab group

Adenine treatment showed a significant (*P* < 0.05) increase in the mRNA expressions of ASK1, JNK, and MAPK compared with the control group. Infliximab treatment showed a significant (*P* < 0.05) decrease in mRNA expression of ASK1, JNK, and MAPK in the curative and the ameliorative groups compared with the diseased group (Fig. 1a–c).

### Histological results

#### Light microscopic results

**Fig. 2:**
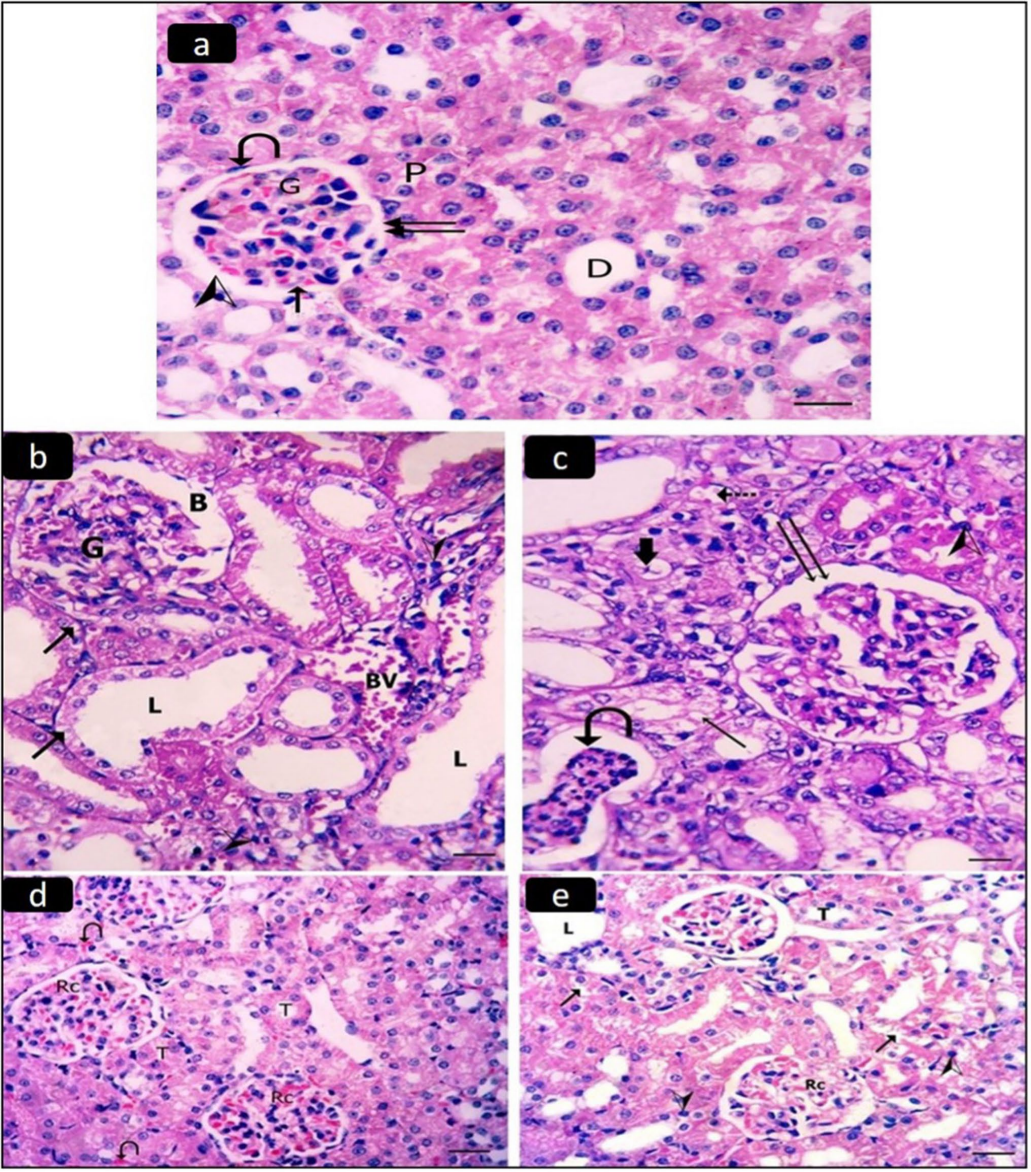
A photomicrograph of a section in the renal cortex of the control group I showing (**a**) Renal corpuscles and tubules. The renal corpuscles (curved arrow) consist of glomeruli (G) surrounded by visceral (arrow) and parietal (double arrows) layers of Bowman's capsule separated by Bowman's space (arrowhead). The cortical renal tubules are formed mainly of proximal and distal convoluted tubules. Proximal convoluted tubules (P) have a relatively small lumen and are lined with large broad cuboidal cells. Distal convoluted tubules (D) have a larger regular lumen and are lined with cuboidal cells (**a**). The diseased group III showing: (**b**) Glomeruli (G) with wide Bowman’s space (B). A large blood vessel (BV) can also be detected. Some tubules are dilated with wide lumina (L). **c)** A segmented glomerulus (double arrow) is seen. Some tubules have desquamated epithelial cells (thick arrow) and homogenous eosinophilic material (knotted arrow) in their lumina. A large deformed tubule is seen with cellular debris in the lumen (curved arrow). Both (**b**) and (**c**): Tubules are deformed with cytoplasmic vacuoles (arrows) in their epithelial lining. Some tubular epithelial cells have small, deeply stained nuclei (arrowhead). **d** The ameliorative group IV showing the normal structure of the renal corpuscle (Rc) and tubules (T). There are some congested blood capillaries between some renal tubules (curved arrow). **e** Curative group V showing intact renal corpuscles (Rc) while some tubules (T) have wide lumina (L). Some tubules have small, deeply stained nuclei (arrowhead) and pale-stained vacuolated cytoplasm (arrow). (H&E, × 400, scale bar 20 µm)

The infliximab group II is nearly the same as the control group I. H&E sections of the control group I renal cortex revealed that it was made up of renal corpuscles and tubules. The glomeruli of the renal corpuscles were surrounded by Bowman’s capsule’s visceral and parietal layers, which were divided by Bowman’s space. The proximal and distal convoluted tubules make up the cortical renal tubules. The lumen of the proximal convoluted tubules was small and lined with large cuboidal cells, whereas the lumen of the distal convoluted tubules was larger and lined with cuboidal cells (Fig. 2a).

H&E sections of the renal cortex of the diseased group III revealed the glomeruli with a wide Bowman’s space. A large blood vessel could also be seen. Some tubules were dilated with wide lumina (Fig. 2b). A segmented glomerulus was seen (Fig. 2c). Tubules were deformed having cytoplasmic vacuoles in their epithelial lining, desquamated epithelial cells, and homogenous eosinophilic material in their lumina. A large deformed tubule was seen with cellular debris in the lumen. The nuclei of certain tubular epithelial cells were small and deeply stained. (Fig. 2b, c).

H&E sections of the renal cortex of the ameliorative group IV showed an intact structure of the renal corpuscle and tubules. There were some congested blood capillaries between some renal tubules. Some interstitial cells were noticed (Fig. 2d). The curative group V showed that some renal tubules had wide lumina. Some tubules had small, deeply stained nuclei and pale-stained vacuolated cytoplasm. There were some interstitial cells (Fig. 2e).

**Fig. 3:**
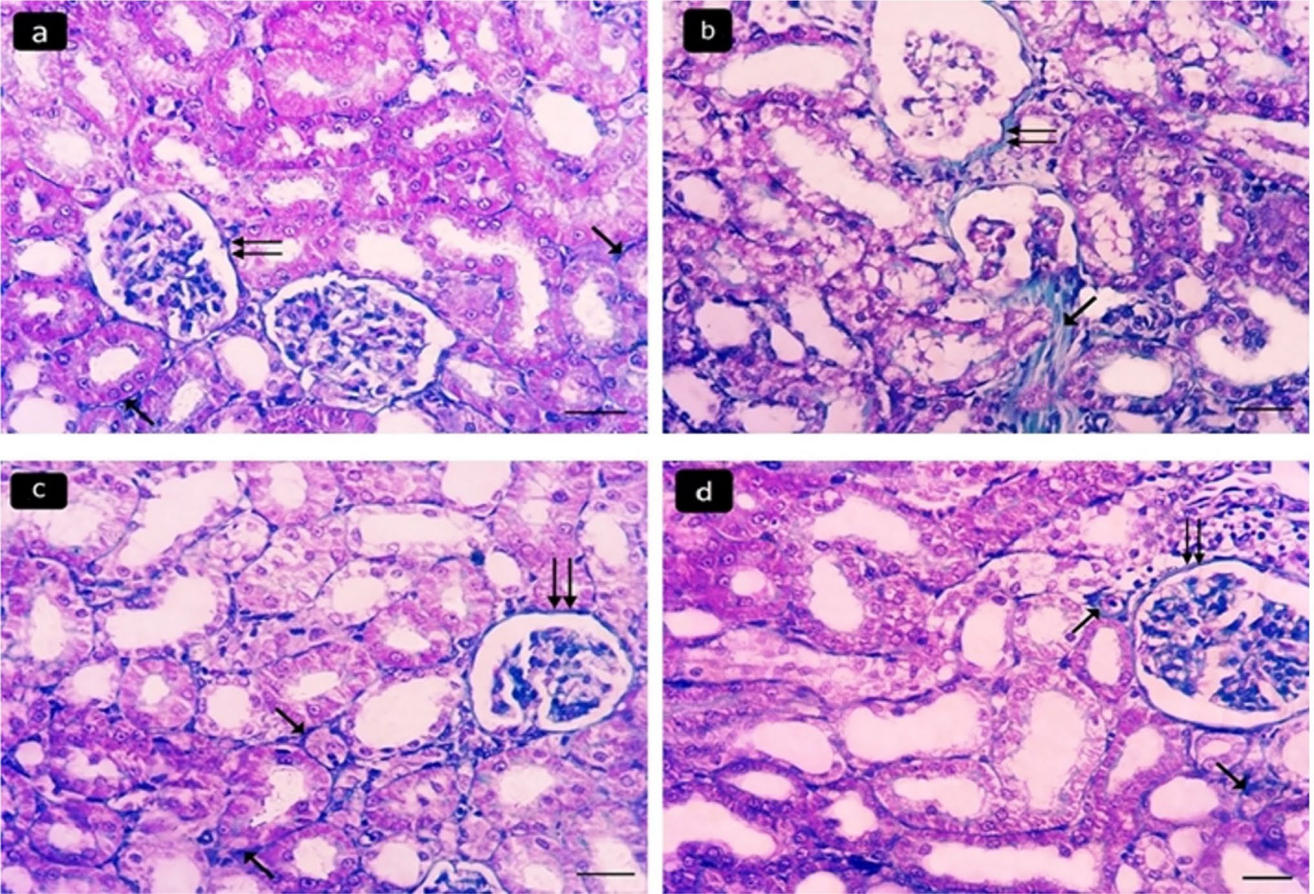
Photomicrographs of Mallory’s trichrome stained sections of the renal cortex of adult male albino rats showing: minimal amount of blue stained collagen fibers around some renal tubules (arrows) and renal glomeruli (double arrows) in control group I (**a**) and ameliorative group IV (**c**). **b** Marked collagen fibers are seen in the diseased group III. **d** Mild collagen fibers are found in the curative group V. Mallory’s trichrome, × 400, scale bar 20 µm)

**Fig. 4:**
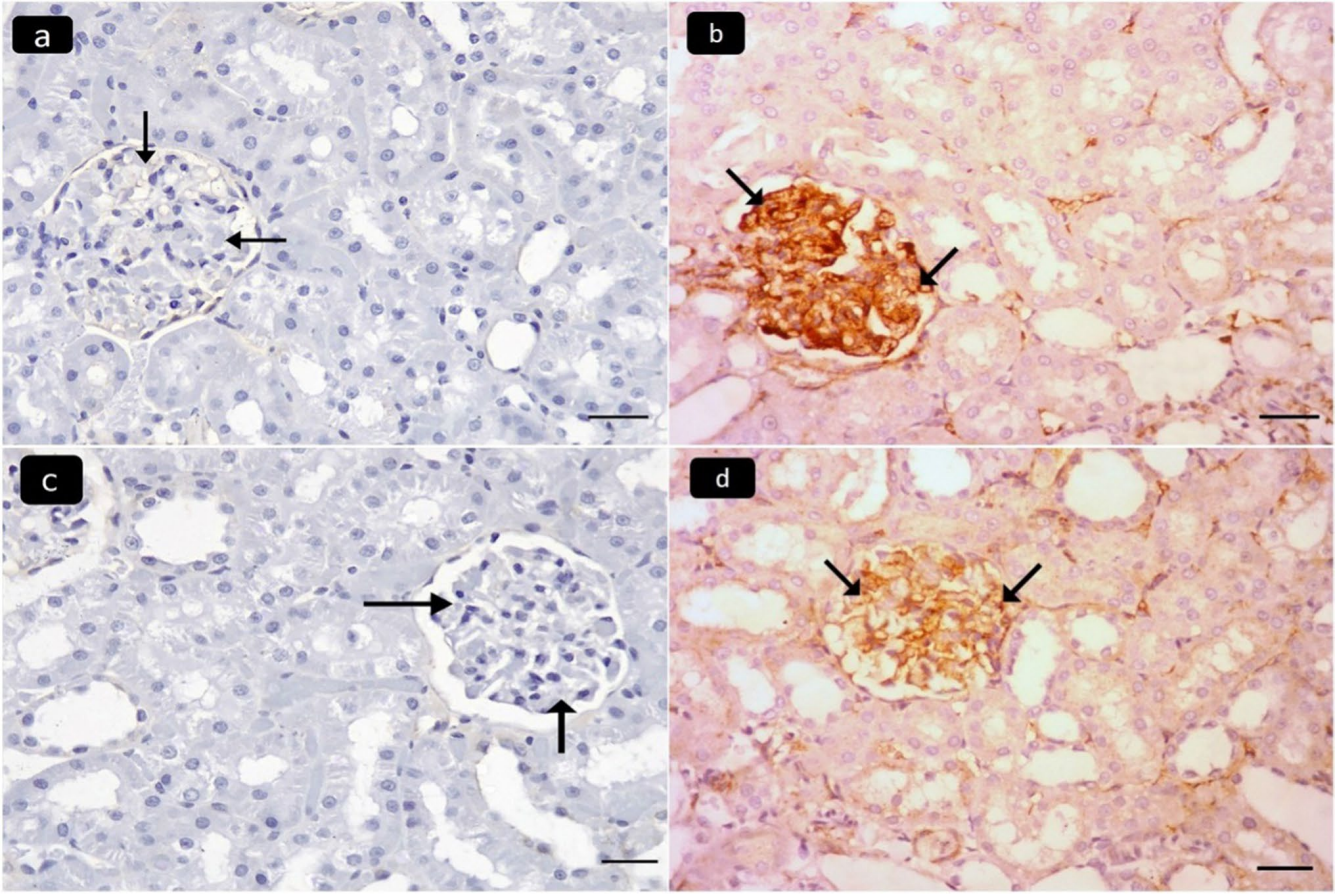
Photomicrographs of stained sections of the renal cortex of adult male albino rats showing: **a** Negative immunostaining of desmin in the cytoplasm of glomerular epithelial cells (arrows) in the Control group I. **b** Strong positive immunostaining in the diseased group III. **c** Minimal immunostaining in the ameliorative group IV. **d** Moderate immunostaining in the curative group V. (Desmin immunostaining, × 400 scale bar 20 µm)

Mallory’s-trichrome stained sections of the renal cortex of the control (I) and the ameliorative (IV) groups revealed a minimal amount of blue-stained collagen fibers around some renal tubules and glomeruli (Fig. 3a, c). The diseased group III showed marked collagen fibers (Fig. 3b). The curative (V) group showed mild collagen fibers (Fig. 3d). Immunohistochemically stained sections of the renal cortex for desmin showed negative immunostaining in the cytoplasm of glomerular epithelial cells in the control group (Fig. 4a), strong positive immunostaining in the diseased group (Fig. 4b), minimal immunostaining in the ameliorative group (Fig. 4c), and moderate immunostaining in the curative group (Fig. 4d).

**Fig. 5:**
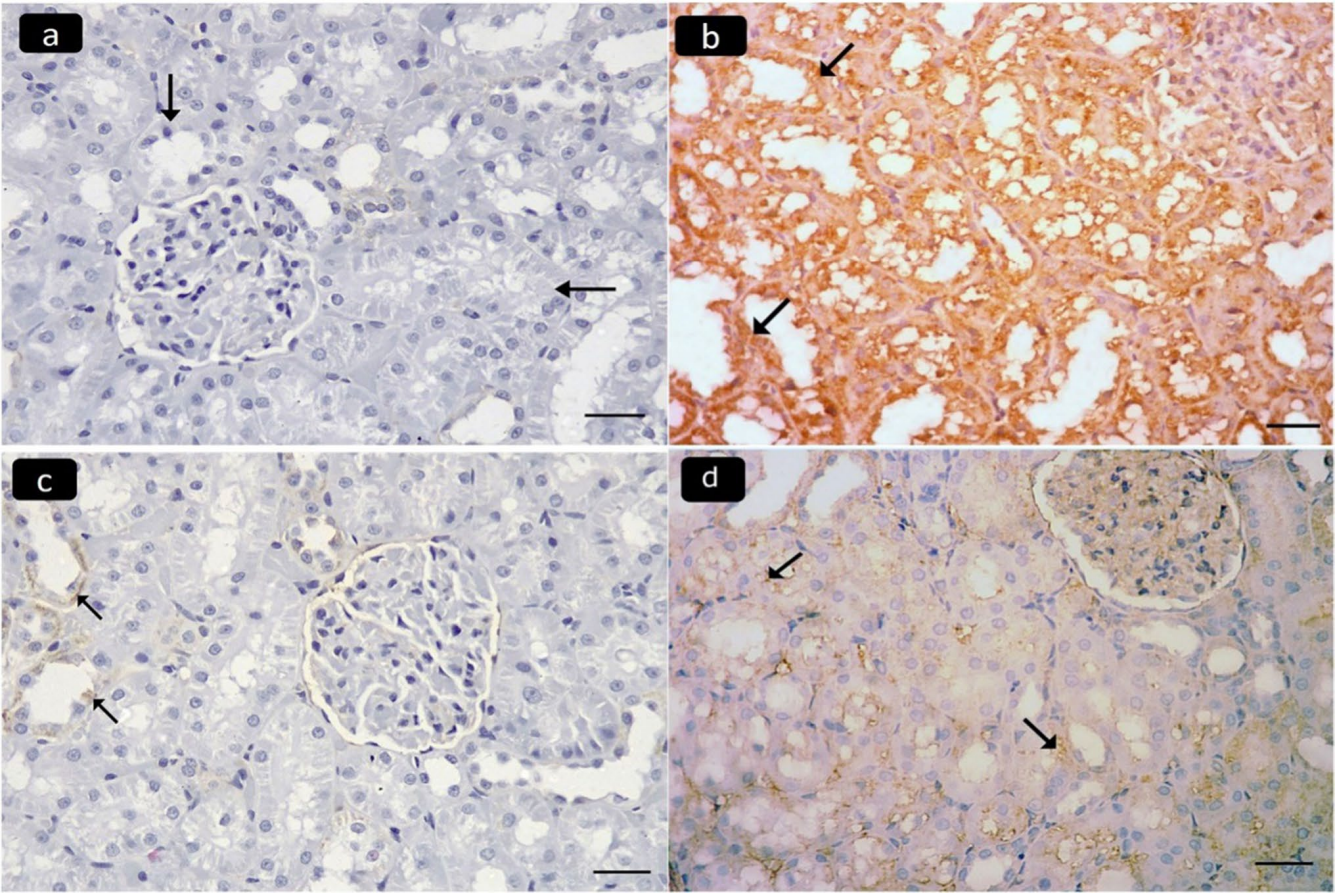
Photomicrographs of stained sections of the renal cortex of adult male albino rats showing: Negative immunostaining of TNF-α in the cytoplasm of renal tubules (arrows) in the Control group I (**a**). **b** Strong positive immunostaining in the diseased group III. **c** Minimal immunostaining in the ameliorative group (IV). **d** Mild immunostaining in the curative group V. (TNF-α immunostaining, × 400 scale bar 20)

Immunohistochemically stained sections of the renal cortex for TNF-α showed negative immunostaining in the cytoplasm of renal tubules in the control group (Fig. 5a), strong positive immunostaining in the diseased group (Fig. 5b), minimal immunostaining in the ameliorative group (Fig. 5c), and mild immunostaining in the curative group (Fig. 5d).

### Morphometric results

#### Area percentage (%) of collagen fibers

Statistical analysis of the area percentage of collagen fiber content showed a very highly significant increase in the diseased group in comparison with the control group. However, in comparison with the control group, the ameliorative group showed a non-significant increase, while the curative group revealed a significant increase (Table 6).

**Table 6:** Effect of infliximab on morphometric and statistical analysis of renal cortex specimens on adenine induced CKD in adult male albino rats

Parameters	Group I (control)	Group II (infliximab)	Group III (diseased)	Group IV (ameliorative)	Group V (curative)
Area percentage of collagen fibers	6.71 ± 0.19	6.91 ± 0.20	20.12 ± 0.58 ^*#^	7.15 ± 0.6 ^*&#^	7.7 ± 0.13 ^*&#^
Area percentage of Desmin immunoexpression	0.4 ± 0.10	0.8 ± 1.20	18.8 ± 1.20 ^*#^	2.9 ± 3.1 ^*&#^	4.2 ± 2.10 ^*&#^
Area percentage of TNF- α immunoexpression	4.12 ± 0.20	5.2 ± 0.80	28.14 ± 4.01 ^*#^	6.6 ± 2.12 ^*&#^	13.07 ± 2.56 ^*&#^

*TNF-α* tumor necrosis alpha

Data represents mean ± SD significant *P*-value < 0.05

^*^Significant to the control group

^&^Significant to the diseased group

^#^Significant to the infliximab group

#### Area percentage (%) of desmin immunoexpression

In a comparison of the diseased group with the control group, statistical analysis of the area percentage of desmin immunoreaction showed a very highly significant increase in the diseased group. However, in comparison with the control group, the ameliorative group showed a non-significant increase, while the curative group exhibited a significant increase (Table 6).

#### Area percentage (%) of TNF-α immunoexpression

When the area % of TNF-α immunoreaction was compared between the diseased and control groups, statistical analysis revealed a highly significant rise in the diseased group. The ameliorative group revealed a non-significant increase compared with the control group. The curative group showed a significant increase compared to the control group (Table 6).

## Discussion

CKD remains a global health issue despite decades of research, with most patients being detected when the disease has progressed to the late stages (Corremans et al. 2022).

Adenine administration, in our recent research, increased the level of urea, creatinine, and NGAL as well as inflammatory (IL-6 and NF-κB), apoptotic (caspase 3), and oxidative stress markers (MDA), TAC was decreased. Also, it increased the expression of AMPK, ASK, and JNK. Furthermore, renal histopathological indicators of destruction (inflammation and fibrosis) were increased. Similar studies have endorsed the same results. Ali et al. (2018) showed that adenine administration for 5 weeks increased urea, creatinine, NGAL, and MDA with decreased TAC. Also, marked histopathological inflammation and fibrosis were detected (Ali et al. 2018). In the same context, Ali et al. (2019) showed that adenine treatment increased IL-6 and TNF-α, which is a notorious inducer of the JNK/ASK pathway (Ali et al. 2019). Intriguingly, Zhang et al. (2018) confirmed that CKD induced with 5/6 nephrectomy caused the upregulation of NF-κB and MAPK (Zhang et al. 2018).

The current study was conducted to consider the effect of infliximab on adenine-induced CKD with insights into its mechanism-based protection. The study demonstrated that infliximab decreased urea, creatinine, and NGAL serum levels, decreased the expression of AMPK, JNK, ASK, and caspase 3. Also, there was a decrease in inflammatory cytokines (IL-6 and NF-κB) levels and oxidative stress markers (MDA) with improvement in histopathological outcomes and TAC level deteriorated by adenine administration.

This study revealed that, concerning urea, creatinine, and NGAL, infliximab declined their serum levels given in a dose of (5 mg/kg, i.p.), and in this context, Saritemur et al. (2015) verified that infliximab decreased the blood level of creatinine and blood urea nitrogen in a rat model of glycerol contrast-stimulated nephropathy (Saritemur et al. 2015). Furthermore, Younis et al. (2021) showed that infliximab reduced the level of urea and creatinine in doxorubicin-induced nephrotoxicity when used as a single dose (5 mg/kg, i.p.) through the inactivation of Wnt/β-catenin/renin-angiotensin axis (Younis et al. 2021). Moreover, Abdelrahman et al. (2020) confirmed that infliximab in a dose of (5 mg/kg, i.p.) decreased the level of NGAL in a rat model of nephrotoxicity caused by doxorubicin (Abdelrahman et al. 2020).

Additionally, as we researched the protective and therapeutic efficacy of infliximab regarding the inflammatory mediators, IL-6 and NF-κB, we found that it decreased their levels and this observation is in line with that of Dadsetan et al. (2016) who found that infliximab in a dose of (5 mg/kg, i.v.) normalized the levels of IL-6 and NF-κB and translocated NF-κB into nucleoli in a rat model of neuroinflammation (Dadsetan et al. 2016).

Furthermore, in our study, the significant elevation in MDA was attenuated by infliximab given in a dose of (5 mg/kg), and interestingly, Triantafillidis et al. (2005) showed that the injection of infliximab with three different doses (5, 10, and 15 mg, s.c.) resulted in a significant decrease in MDA level in a rat model of colitis induced with 2,4,6, trinitrobenzene sulfonic acid (Triantafillidis et al. 2005). Additionally, infliximab administration showed higher TAC, and this agreed with Abdelrahman et al. (2020), who showed that infliximab administration in a dose of (5 mg/kg, i.v.) increased TAC in a rat model of nephrotoxicity induced by doxorubicin (Abdelrahman et al. 2020).

Additionally, a reduction in the level of caspase 3, the crucial mediator of programmed cell death, with infliximab administration was a prominent finding, and this is in agreement with that of Saritemur et al. (2015), who proved that infliximab administration at a dose of (5 mg/kg, i.p.) decreased caspase 3 expression in a rodent model of glycerol-contrast-induced nephropathy (Saritemur et al. 2015).

TNF-induced ASK1 activation was discovered to be regulated by tumor necrosis factor-receptor-associated factors (TRAFs), which are critical in the regulation of ASK1 activity (Nishitoh et al. 1998), and as infliximab decreased TNF-α level (Cheng et al. 2014) it could, as a result, decrease ASK activation. Moreover, infliximab treatment caused decreased expression of MAPK in our model, and this agreed with that of Kato et al. (2013), who studied the role of P38/MAPK in sciatic nerve injury and confirmed that there is a positive feedback loop between TNF-α and p38 MAPK in which TNF-α phosphorylates p38 MAPK, leading to increased TNF-α production in the same cell type, and as an anti-TNF, infliximab could inhibit MAPK phosphorylation (Kato et al. 2013).

Histopathological results indicated renal damage in the adenine-treated group. H&E-staining sections revealed deformed segmented glomeruli with broad Bowman’s space and dilated blood vessels. Small, darkly pigmented nuclei, and cytoplasmic vacuoles were found in the dilated and deformed renal tubules. These results agreed with Kim et al. (2010), who confirmed that oxidative stress and inflammation are involved in the renal failure model induced with adenine (Kim et al. 2010). Renal tubular damage could be caused by oxidative stress and inflammation accompanying adenine administration (Malek and Nematbakhsh 2015; Gong et al. 2019).

In addition, in the lumina of some renal tubules, a homogeneous eosinophilic material was observed. Pisoni et al. (2008) defined it as a cast, explaining that it was formed by exfoliating necrotic epithelial cells, which provided a favorable matrix for cast formation (Pisoni et al. 2008).

In contrast, an improvement was observed mainly in the ameliorative group taking infliximab concomitant with adenine for 5 weeks and to some extent in the curative group taking infliximab as a single dose after 5 weeks as the preserved structure of renal corpuscles and tubules. This was explained by Tasdemir et al. (2012), who stated that infliximab prevents some tissue injuries by inhibiting TNF-α and decreasing the formation of reactive oxygen species (ROS) (Tasdemir et al. 2012).

This study used Mallory’s trichrome stain to detect fibrosis. Collagen deposition was observed inside the glomeruli and between the renal tubules in the sections of the adenine-treated group. Inflammatory cells and chemokines such as transforming growth factor-beta 1 (TGF-β1) stimulate myofibroblasts, which are responsible for developing extracellular matrices such as collagen and fibronectin, and hence play a role in renal interstitial fibrosis. Additionally, TNF-α causes interstitial cells to convert into myofibroblasts, resulting in collagen deposition (Farris and Colvin 2012).

Furthermore, an improvement was observed in the ameliorative and curative groups in the form of minimal and moderate fibrosis, and this was in line with Elzbieta et al. (2008), who established the idea that inactivating TNF-α with infliximab reduces TGF-β (Elzbieta et al. 2008).

Desmin, an intermediate filament protein, has been proposed as an indicator for podocyte injury, and its expression has usually been elevated in glomerular disorders involving podocyte damage (Qin et al. 2012). In this context, Haiting et al. (2017) reported that TGF-β1, which is produced by glomerulosclerosis, increases the expression of desmin and caspase 9, which leads to apoptosis (Haiting et al. 2017).

Fascinatingly, immunohistochemical results revealed a distinct increase in desmin immunoexpression in the renal corpuscles of the adenine-treated group. This is in line with Walaa et al. (2019), who noticed an increase in desmin immunoexpression with bisphenol nephrotoxicity (Walaa et al. 2019). On the other side, there was a decrease in desmin immunoexpression in the renal corpuscles of the renal cortex with concomitant administration of infliximab, the finding, which is in line with that of Eto et al. (2007), who stated that the decrease in desmin protein expression plays a major role in regulating injured podocytes (Eto et al. 2007).

Additionally, immunohistochemical results revealed an increase in TNF-α immunoexpression in the renal tubules of the group treated with adenine, and these results were detected by Elenkov et al. (2005), who claimed that adenine-induced chronic renal failure induced the discharge of pro-inflammatory cytokines such as IL-6 and TNF-α (Elenkov et al. 2005). On the other side, there was a decrease in TNF-α immunoexpression in the renal tubules of the renal cortex with concomitant administration of infliximab, and this agreed with Tasdemir et al. (2012), who confirmed that infliximab inhibits the expression of TNF***-***α (Tasdemir et al. 2012).

## Conclusion

Infliximab (5 mg/kg; i.p.) abated oxidative stress, inflammation, and apoptosis in adenine-fed rats, given as an ameliorative agent. Also, infliximab (5 mg/kg; i.p.) showed a curative effect given as a single dose after adenine treatment.

Infliximab could be indicated as a possible adjuvant therapy in chronic renal disease owing to its high anti-inflammatory and antioxidant effects demonstrated in this study, but more investigations will be needed to confirm this impact.

### Supplementary Information

Below is the link to the electronic supplementary material.

Supplementary file1 (ZIP 38 kb)

## Data Availability

The datasets created during this investigation are available upon request from the corresponding author.
